# The efficacy of Chinese herbal medicine on anxiety and depression in patients with chronic prostatitis complicated by sexual dysfunction: a systematic review and meta-analysis protocol

**DOI:** 10.3389/fpsyt.2025.1632159

**Published:** 2025-08-05

**Authors:** Zhaozhan Xie, Xuecheng Zhang, Bai Li, Qi Wang, Zhihui Hou, Hongling Jia, Yongchen Zhang

**Affiliations:** ^1^ Department of Acupuncture and Moxibustion, Second Affiliated Hospital of Shandong University of Traditional Chinese Medicine, Jinan, Shandong, China; ^2^ School of Acupuncture-Moxibustion and Tuina, Shandong University of Traditional Chinese Medicine, Jinan, Shandong, China; ^3^ Department of Proctology, China-Japan Friendship Hospital, Beijing, China; ^4^ Second School of Clinical Medicine, Shandong University of Traditional Chinese Medicine, Jinan, Shandong, China

**Keywords:** Chinese herbal medicine, anxiety, depression, chronic prostatitis, sexual dysfunction, systematic review

## Abstract

**Background:**

Chronic Prostatitis (CP) is often accompanied by urinary symptoms such as dysuria and frequency, as well as sexual dysfunction including erectile dysfunction and reduced libido. Men with CP are at a significantly greater risk of developing anxiety and depression compared to healthy controls. Chinese Herbal Medicine (CHM) provides a holistic approach by simultaneously targeting inflammation, pelvic microcirculation, and neuropsychiatric pathways, which aligns with the multifactorial nature of CP. Therefore, this study will conduct a systematic review and meta-analysis to evaluate and summarize the efficacy of CHM for managing anxiety and depression in patients with CP complicated by sexual dysfunction.

**Methods:**

This research will conduct a systematic search of four Chinese databases (China National Knowledge Infrastructure, Wanfang Database, China Biomedical Database, and VIP Database) as well as four international databases (PubMed, Web of Science, EMBASE, and Cochrane Library). It will focus on identifying randomized controlled trials (RCTs) that examine the effects of CHM interventions for CP complicated by sexual dysfunction and co-occurring anxiety and depression. The selected studies will then be subject to thorough screening and quality evaluation using standardized instruments.

**Results:**

The results of this study will provide a reliable basis for the efficacy of CHM for managing anxiety and depression in patients with CP complicated by sexual dysfunction.

**Conclusions:**

This study will compare the clinical efficacy of CHM in managing anxiety and depression in patients with CP complicated by sexual dysfunction, thereby assisting clinicians and patients in selecting more effective intervention regimens during the clinical decision-making process.

**Systematic review registration:**

https://www.crd.york.ac.uk/prospero/, identifier CRD420251002787.

## 1 Introduction

Prostatitis, a heterogeneous group of inflammatory conditions affecting the prostate gland, is classified into four subtypes—acute bacterial prostatitis, chronic bacterial prostatitis, chronic prostatitis (CP), and asymptomatic inflammatory prostatitis—according to the National Institutes of Health (NIH) classification system (1). Among these, CP—accounting for approximately 90% of cases, often accompanied by urinary symptoms such as dysuria and frequency, as well as sexual dysfunction including erectile dysfunction and reduced libido (2). Emerging evidence suggests a multifactorial pathophysiology, involving neurogenic inflammation, pelvic floor myofascial dysfunction, autoimmune responses, and dysregulation of the hypothalamic-pituitary-adrenal (HPA) axis (3). Pro-inflammatory cytokines, including TNF-α, IL-6, and IL-8, are elevated in expressed prostatic secretions, contributing to chronic tissue damage and peripheral sensitization (4, 5). Furthermore, the cross-talk between pelvic nerves and the central nervous system (CNS) exacerbates pain perception via mechanisms involving glutamate signaling and microglial activation (6, 7). Globally, the prevalence of prostatitis varies between 2% and 10%, and approximately 35–50% of patients experience concomitant sexual dysfunction (8–10). Population-based studies have demonstrated that men with CP are at a significantly greater risk of developing anxiety and depression compared to healthy controls (11). This bidirectional relationship arises from chronic pain disrupting dopaminergic and serotonergic pathways that are critical for mood regulation, while psychological distress reduces pain thresholds through HPA axis hyperactivity and glucocorticoid resistance (12, 13). Sexual dysfunction further exacerbates this burden, as erectile dysfunction and premature ejaculation are associated with diminished self-esteem, social withdrawal, and impaired quality of life (14). First-line treatments for CP include α-blockers, anti-inflammatory agents, and antibiotics; however, their efficacy remains suboptimal due to the heterogeneity of underlying etiologies (15, 16). A Cochrane review concluded that α-blockers provide only modest symptomatic relief in 25–30% of patients and do not significantly improve sexual function (17). For comorbid anxiety and depression, selective serotonin reuptake inhibitors (SSRIs), such as sertraline, are commonly prescribed; however, these medications frequently exacerbate sexual dysfunction, including anorgasmia and reduced libido (18, 19). Psychological interventions, such as cognitive-behavioral therapy (CBT), show promise; however, their effectiveness is limited by issues of accessibility and cost (20, 21).

Chinese herbal medicine (CHM) provides a holistic approach by simultaneously targeting inflammation, pelvic microcirculation, and neuropsychiatric pathways, which aligns with the multifactorial nature of CP. For instance, Gardenia jasminoides, a traditional Chinese herb, contains geniposide, a compound that inhibits NF-κB and MAPK signaling pathways and attenuates prostatic inflammation in mouse models (22, 23). Similarly, the herbal combination of Bupleurum chinense DC (Chaihu) and Paeonia lactiflora Pall (Baishao) exerts sustained antidepressant effects *in vivo*, mediated through antioxidant pathway activation, HPA axis homeostasis modulation, and neural synaptic plasticity preservation mechanisms (24). Clinical trials have demonstrated that CHM significantly improves International Prostate Symptom Scores (IPSS) and quality-of-life (QoL) metrics compared to placebo (25, 26). Furthermore, CHM formulations lack the sexual side effects commonly associated with SSRIs and can effectively alleviate symptoms of anxiety and depression (18, 27, 28).

Despite growing evidence, no systematic review has yet synthesized the dual efficacy of CHM on both urogenital and psychological outcomes in this population. Existing meta-analyses either narrowly focus on pain relief or exclude studies that address sexual dysfunction. This review will address critical gaps by evaluating the effects of CHM on anxiety and depression using validated scales, analyzing subgroup differences according to the severity of sexual dysfunction, and comparing the safety profile of CHM with that of conventional therapies. The findings are anticipated to offer clinical insights into integrating CHM into multidisciplinary care models for CP. Therefore, our team will conduct a meta-analysis and systematic review to evaluate and summarize the efficacy of CHM in managing anxiety and depression among patients with CP complicated by sexual dysfunction.

## 2 Materials and methods

This study will be conducted according to the Preferred Reporting Item (PRISMA) for systematic reviews and meta-analyses (29, 30). The registration number of the protocol is CRD420251002787 (https://www.crd.york.ac.uk/prospero/).

### 2.1 Inclusion and exclusion criteria

#### 2.1.1 Type of study

The selection process strictly adhered to RCTs as the sole admissible study design, while restricting the search to English and Chinese language publications. Methodological exclusion criteria encompassed non-randomized studies, including but not limited to observational research paradigms, cross-sectional survey methodologies, and preclinical animal experimentation.

#### 2.1.2 Type of participants

This study will only include participants with anxiety and depression among those diagnosed with CP complicated by sexual dysfunction. Participants of age, occupation, education level, or severity will be considered. Nevertheless, patients with a psychiatric disorder or a history of taking psychotropic medications will be excluded.

#### 2.1.3 Type of intervention

The experimental group must receive the CHM intervention. CHM interventions are classified into: Monotherapy (single-herb preparations); Standard combination therapy (classical formulas with ≤8 herbs); Complex combination therapy (customized formulas with >8 herbs or integrated modalities). While the control group may receive: (a) Placebo control; (b) No treatment; (c) Drug therapy specifically for anxiety, depression, sexual dysfunction or CP (e.g., anxiolytics like benzodiazepines, antidepressants like SSRIs, PDE5 inhibitors like sildenafil for erectile dysfunction, or α-receptor blocker like Tamsulosin Hydrochloride Sustained-release Capsules for CP); (d) Psychological counseling. Including varied controls facilitates a broad assessment of CHM efficacy.

#### 2.1.4 Type of outcome measures

Outcome measures required at least one assessment of anxiety, depression, or overall symptoms. Primary outcomes included the Self-Rating Anxiety Scale (SAS) (31), the Self-Rating Depression Scale (SDS) (32), the Hamilton Anxiety Rating Scale (HAMA) (33), the Hamilton Depression Rating Scale (HAMD) (34), the Beck Anxiety Inventory (BAI) (35), the Beck Depression Inventory (BDI) (36), and the Depression Anxiety Stress Scales (DASS) (37). Secondary outcomes included intravaginal ejaculation latency time (IELT) scores (38), International Index of Erectile Function-5 (IIEF-5) scores (39), and Prostatitis-specific Quality of Life Scale (Pro-QOL) (40).

### 2.2 Search strategy

Two researchers will comprehensively search four Chinese databases, including China National Knowledge Infrastructure (CNKI), Wanfang Data Knowledge Service Platform, VIP, and CBM, and four English databases, including PubMed, Web of Science, EMBASE, and The Cochrane Library. We will search the databases for all articles from their inception to 20 May 2025 in Chinese and English, with no geographical restrictions. A combination of subject matter and free terminology will be employed to ensure a comprehensive search, regardless of language or type of publication. All databases will be searched to ensure that all relevant articles will be identified. Appendix S1 provides a timeline of the review process. **Table 1** provides a sample search strategy for the PubMed database.

**Table 1:** The search strategy for PubMed.

ORDER	STRATEGY
#1	Chronic Disease[Mesh] OR Prostatitis[Mesh]
#2	Premature Ejaculation[Mesh] OR Erectile Dysfunction[Mesh] OR male sexual dysfunction[Title/Abstract] OR Dysfunction, Erectile[Title/Abstract]
#3	Anxiety[Mesh] OR Depression[Mesh] OR Nervousness[Title/Abstract] OR Anxiousness[Title/Abstract] OR Depressive Symptom[Title/Abstract]
#4	Medicine, Chinese Traditional[Mesh] OR Chinese herbal medicine[Title/Abstract] OR herbal[Title/Abstract]
#5	randomized controlled trial[Publication Type] OR randomized[Title/Abstract] OR placebo[Title/Abstract]
#6	#1 AND #2 AND #3 AND #4 AND #5

### 2.3 Study selection

**Figure 1:**
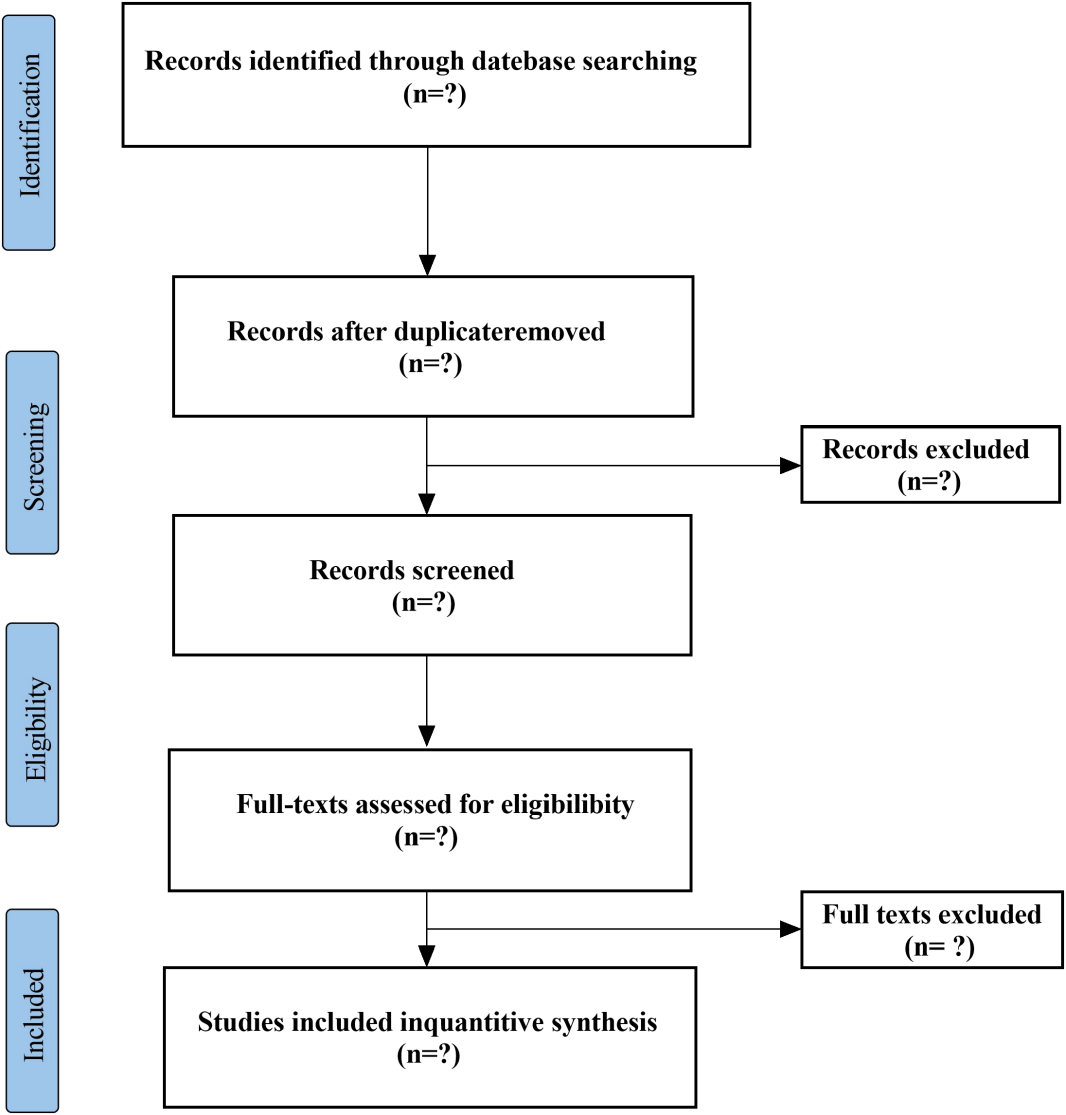
The PRISMA flow diagram of the study selection process.

Two researchers will independently screen the titles and abstracts to determine their eligibility based on the predefined inclusion criteria and will subsequently conduct full-text evaluations of all studies that meet these criteria. The following data will be systematically extracted using standardized forms: the first author’s name, year of publication, age range of participants, sample size, diagnostic criteria, interventions in both the treatment and control groups, duration of treatment, outcome measures, and adverse events. In cases where disagreements arise, a third researcher will be consulted, and a consensus will be reached through discussion. Duplicate manuscripts will be identified by matching authors, sample sizes, and outcomes. For overlapping data, we will: include only the most complete dataset; contact authors if unclear; perform sensitivity analyses excluding suspected duplicates. The PRISMA flowchart (**Figure 1**) will provide a clear and comprehensive overview of the study selection process.

### 2.4 Assessment of risk of bias in included studies

Two evaluators will independently analyze the potential bias risk for every included randomized controlled trial (RCT) by utilizing the Cochrane Handbook’s recommended Risk of Bias tool, RoB 2.0 (41). This analysis will focus on several key areas: generation of random sequences, concealment of allocation, masking of participants and staff, blinding of outcome assessors, missing outcome data, selective disclosure, and additional possible sources of bias. Meanwhile, we will explicitly evaluate the plausibility of blinding in light of CHM’s sensory properties. With regard to intervention fidelity, data will be extracted on the following aspects: quality control measures for CHM; standardization of decoction procedures; and methods for adherence monitoring. Following this evaluation, each study will be categorized as exhibiting a high, low, or unclear level of bias risk. Should any discrepancies arise, a third evaluator will be involved to help achieve agreement.

### 2.5 Dealing with missing data

Missing standard deviations (SDs) will be imputed using the following hierarchy: Contact authors via email twice at 2-week intervals; Calculate from p-values/confidence intervals; Impute using the maximum SD from comparable studies. Skewed data will be log-transformed prior to pooling. A modified analysis will then be conducted using the available data, and we will consider the potential impact of any missing information on our findings.

### 2.6 Data synthesis

A meta-analysis will be conducted using STATA. Since each score is a numeric variable that may involve different measurement scales and scoring systems, we will calculate the differences in scores before and after treatment. The standardized mean difference (SMD) will be chosen as the effect size measure, with 95% confidence intervals (95% CI) used to define the statistical range of effect estimates. Heterogeneity among studies will be assessed using the Chi-squared test and I² statistic. If heterogeneity is low (I² < 50%), a fixed-effect model will be applied for the meta-analysis. In cases of significant heterogeneity (I² ≥ 50%), a random-effects model will be adopted, followed by subgroup analyses to explore potential sources of heterogeneity. When quantitative synthesis is not feasible, a narrative summary of the results will be provided for included publications. For trials that only report pre- and post-intervention values, mean changes will be determined by subtracting baseline values from post-intervention measurements, with corresponding standard deviations of change estimated appropriately.

### 2.7 Subgroup analysis

If substantial heterogeneity is identified, we will carry out subgroup analyses to explore possible causes of variability. Pre-specified subgroup factors: CHM formulation (Decoction/Capsule/Granule); Treatment duration (<8 weeks/≥8 weeks); CP severity (NIH-CPSI mild/moderate/severe); Geographic region (Eastern Asia vs. other); Concomitant therapy (CHM monotherapy vs. CHM+conventional). When sufficient data are available across subgroups, mixed-effects models will be applied for quantitative analyses to assess interactions between subgroups and treatments. For subgroups with inadequate data for quantitative synthesis, a systematic qualitative synthesis will be conducted instead. This will include within-study comparisons, cross-study pattern evaluations, and evidence rating using the GRADE approach, allowing for insightful interpretation without relying on formal meta-analytic techniques (42).

### 2.8 Sensitivity analysis

If significant heterogeneity remains after conducting subgroup analyses, we will perform sensitivity analyses to assess the stability of the results. The sensitivity analysis procedure will entail systematically re-executing the meta-analysis while omitting studies identified as having a high risk of bias (RoB ≥ 4 on the modified Newcastle-Ottawa Scale) and statistical outliers detected via Galbraith plots. By comparing effect size estimates from the primary analysis with those obtained in the sensitivity analysis using the Hartung-Knapp adjustment, we will measure the impact of individual studies on the overall pooled effects. This methodological strategy allows for an evaluation of result reliability while identifying potential sources of heterogeneity through the differential effects of exclusions.

### 2.9 Publication bias

If the meta-analysis comprises 10 or more studies, we will assess publication bias by applying Egger’s regression test. Additionally, funnel plots will be used to visually examine any potential asymmetry in the distribution of effect sizes.

## 3 Discussion

This protocol outlines a systematic review and meta-analysis designed to explore the efficacy of CHM in alleviating anxiety and depression among patients with CP complicated by sexual dysfunction. By evaluating published RCTs, we will comprehensively assess the impact of CHM interventions on both urogenital symptoms and psychological outcomes. Furthermore, we aim to investigate the potential associations between CHM treatment characteristics and clinical efficacy to identify the optimal therapeutic regimen. The findings are anticipated to bridge the knowledge gap regarding CHM’s holistic effects in CP management and inform evidence-based integration of herbal therapies into multimodal treatment strategies. It is worth noting that inherent ethical challenges in CHM trials (e.g., informed consent, risk-benefit assessment) will be examined across the included RCTs. The absence of ethics committee approval or documentation of informed consent will be recorded as an indicator of study quality.

However, this systematic review and meta-analysis have several limitations. First, although rigorous inclusion criteria were applied to ensure study quality, this approach may have excluded relevant clinical data from smaller-scale trials or observational studies, thereby limiting the scope of eligible evidence on CHM for managing anxiety, depression, and sexual dysfunction in CP patients. While RCTs are prioritized for efficacy assessment due to minimized confounding, we recognize that real-world evidence (e.g., observational studies) may provide complementary insights on CHM safety and long-term outcomes. Given our focus on quantifying causal effects, RCTs remain the gold standard. Future updates could expand to observational designs once methodological standards for CHM real-world data mature. Second, restricting the analysis to English- and Chinese-language publications introduces potential language-based publication bias, as negative results or regional herbal formulations reported in other languages were not considered. Nevertheless, empirical evidence confirms that excluding non-indexed languages with limited CHM literature (e.g., Korean, Japanese) does not significantly alter conclusions, and rigorous screening of reference lists will mitigate publication bias. Third, our analysis primarily focused on commonly prescribed CHM interventions but did not systematically evaluate rare herbal combinations or traditional decoction methods, which may affect the generalizability of efficacy and safety conclusions across diverse CHM practices.

Despite these limitations, this study synthesizes the current evidence on CHM’s dual role in alleviating both psychological distress and urogenital symptoms in CP populations and underscores the need for standardized outcome reporting in future trials. These findings could inform the design of pragmatic, culturally adapted CHM regimens for complex CP cases.
