# Supplementary material for: The efficacy of Chinese herbal medicine on anxiety and depression in patients with chronic prostatitis complicated by sexual dysfunction: a systematic review and meta-analysis protocol
Source: Front Psychiatry. 2025 Aug 5;16:1632159. doi: 10.3389/fpsyt.2025.1632159 (PMC12362715; doi:10.3389/fpsyt.2025.1632159)
Supplement: Supplementary file 1 [file Table1.docx]

S1 The timeline of the review process

| **Phase** | **Timing** |
| --- | --- |
| Database Search | May-Sep 2025 |
| Full-text screening | Oct-Dec 2025 |
| Analysis | Jan-Mar 2026 |
| Manuscript finalization | Apr-Jun 2026 |
